# New Trade Products

**Published:** 1906-11-24

**Authors:** 


### NEW TRADE PRODUCTS.

### RAMIE FABRICS: THEIR SUITABILITY AS CLOTHING.

(Messrs. J. Spencer, Turner Co., of 13 Jewin Crescent, London, E.C.)

This firm has kindly submitted to us woven specimens of cloth made from this important fabric, showing how Ramie can be adapted to everyday use as garments—suitable both for dress materials and for underwear, but particularly suitable for clothing intended for tropical use. This product has been long known to Asiatics as being derived from China grass. The difficulty hitherto met with in this country has been the absence of suitable machinery whereby the sliver—that is, the silky-looking down found in the grass—can be properly prepared and spun into yarn. In this country no adequate machinery has yet been found on any large scale quite suitable for decorticating Ramie, and hence most of the yarns used in the fabrics submitted to us have been imported from Germany. Given new machinery and a plentiful supply of the China grass, which it may be said grows wild in most of our own Colonies, it will be possible, in the hands of the cotton manufacturers who are ready and willing to largely utilise the prince of fabrics, to create what may be called a new industry, not interfering with but, on the other hand, inducing a tendency to enhance and enliven the allied industry of cotton-spinning.

The Germans have unquestionably been ahead of this country in devising machinery for preparing Ramie and in recognising the value of the fabric as allied to other fabrics like silk, cotton, and linen, but as being a more serviceable and superior product in many respects to either the fibre of cotton or of linen. Its superiority consists in the beauty of the woven texture, its silky sheen, and its elasticity, not met with in cotton fabrics, and from the fact that it may be made up into garments as easily as the better known fibres. There is at present in this country no organisation to encourage the manufacture of these goods or to provide for a steady delivery of the raw material. And yet the manufacturers in Manchester would gladly avail themselves of an organisation of this kind capable of providing them with steady supplies of sliver, if possible at a lower price than at present obtains. Such an industry would be of immense value to all our Colonies if this product became better known and the garments made from it became fashionable, being as they are appropriate for wear not only at home but in all Colonial countries. Ramie can be prepared in a raw state unbleached and manufactured into cloth textures so strong and durable as to make it of inestimable value in providing material to form the inner lining of pneumatic tyres of motor-cars and other vehicles. In thick layers it is used in other industries for filter purposes where a filter has to withstand enormous pressure, the fibres being so strong as to enable great strain to be endured. When prepared by delicate machinery it can be woven into silk-like yarns and form the dainty under-garments of ladies with the most delicate and sensitive skins. Its tendency is to keep the body at an equable and uniform temperature, giving comfort and warmth, without undue weight and bulk—very desirable qualities in the underclothing of ladies. It is woven by the firm in question into all sorts of textures. It readily takes up the favourite dyes and retains them. Its washable qualities render it capable of being thoroughly cleansed, making garments made from it differ in this respect very much from woollen fabrics which absorb excreted cutaneous débris and can hardly be cleansed without in some way injuring the fabric. Scarcely any amount of vigorous laundry work will cause the destruction of the Ramie fabric. The fabric has long been in use in the East, but it is fair to add that only in the light of our present-day knowledge and with adequate machine equipment that the highest class of fabrics, such as those submitted to us, can be made possible.

---

The treasurers of the Middlesex Hospital have received a further donation of fifty guineas from the Worshipful Company of Merchant Taylors.

